# Using ZINC08918027 inhibitor to determine Aurora kinase-chromosomal passenger complex isoforms in mouse oocytes

**DOI:** 10.1186/s13104-022-05987-4

**Published:** 2022-03-07

**Authors:** Caroline Kratka, David Drutovic, Cecilia S. Blengini, Karen Schindler

**Affiliations:** 1 Department of Genetics, Rutgers University, The State University of New Jersey, 145 Bevier Rd, Piscataway, NJ 08854 USA; 2 Laboratory of DNA Integrity, Institute of Animal Physiology and Genetics of the Czech Academy of Sciences, Rumburska 89, Libechov, Czech Republic; 3 Human Genetics Institute of NJ, Piscataway, NJ USA

**Keywords:** Aurora kinase, Oocyte, Meiosis, Chromosomal passenger complex

## Abstract

**Objective:**

Miscarriages affect 10% of women aged 25–29, and 53% of women over 45. The primary cause of miscarriage is aneuploidy that originated in eggs. The Aurora kinase family has three members that regulate chromosome segregation. Therefore, distinguishing the roles of these isoforms is important to understand aneuploidy etiology. In meiosis, Aurora kinase A (AURKA) localizes to spindle poles, where it binds TPX2. Aurora kinase C (AURKC) localizes on chromosomes, where it replaces AURKB as the primary AURK in the chromosomal passenger complex (CPC) via INCENP binding. Although AURKA compensates for CPC function in oocytes lacking AURKB/C, it is unknown whether AURKA binds INCENP in wild type mouse oocytes. ZINC08918027 (ZC) is an inhibitor that prevents the interaction between AURKB and INCENP in mitotic cells. We hypothesized that ZC would block CPC function of any AURK isoform.

**Results:**

ZC treatment caused defects in meiotic progression and spindle building. By Western blotting and immunofluorescence, we observed that activated AURKA and AURKC levels in ZC-treated oocytes decreased compared to controls. These results suggest there is a population of AURKA-CPC in mouse oocytes. These data together suggest that INCENP-dependent AURKA and AURKC activities are needed for spindle bipolarity and meiotic progression.

**Supplementary Information:**

The online version contains supplementary material available at 10.1186/s13104-022-05987-4.

## Introduction

The conserved serine/threonine protein kinases in the Aurora kinase (AURK) family play critical roles in regulating chromosome segregation in many cell types [1, 2]. Unlike non-mammalian eukaryotes which encode one or two *Aurk* homologs, the mammalian genome encodes three [3, 4]. AURKA and AURKB isoforms are expressed in both mitotic and meiotic cells, whereas the AURKC isoform is expressed mainly in meiotic cells. In mouse oocytes, AURKA localizes to spindle poles and microtubules by binding its activator, TPX2 [5, 6] where it is important for normal spindle formation [7]. AURKC is the catalytic subunit of the meiotic Chromosomal Passenger Complex (CPC), where it binds the scaffolding unit INCENP (Inner Centromere Protein) and localizes to chromatin. AURKC is responsible for most functions that AURKB-CPC has in mitosis [8, 9], including regulating chromosome alignment and correcting erroneous kinetochore-microtubule attachments [10–12]. As a result, AURKB is diffuse in the oocyte cytoplasm and has still to be defined mechanisms in protecting egg quality with age [13, 14]. Therefore, subcellular localization and function of AURKA and AURKC in mouse oocytes is dictated by their binding to different activators as in mitotic cells.

Analyses of oocyte-specific mouse knockout strains reveals complex genetic interactions amongst the kinases that appear to be unique to the female germline. For example, AURKC negatively regulates AURKA by competing for INCENP binding thereby promoting a spindle length necessary for successful asymmetric cell division [15]. In oocytes lacking AURKC, there is an INCENP-associated population of AURKA that compensates, and it is detectable by microscopy. In HeLa cells, AURKA co-immunoprecipitates with INCENP although it cannot be detected on chromosomes by immunocytochemistry [16]. These findings prompted us to consider whether there was a subpopulation of INCENP-bound AURKA in wild-type (WT) mouse oocytes that is undetectable by available methods of protein visualization.

To more accurately characterize AURK complexes, a tool that disrupts binding partner interactions rather than catalytic activity is necessary. The activator binding site inhibitor, ZINC08918027 (ZC), blocks the AURK::INCENP interaction by affinity for the INCENP binding site on the kinase in mitotic cells [17]. Here we examine the utility of ZC in disrupting CPC function in mouse oocytes.

## Main text

### Materials and methods

#### Mice

**Fig. 1:**
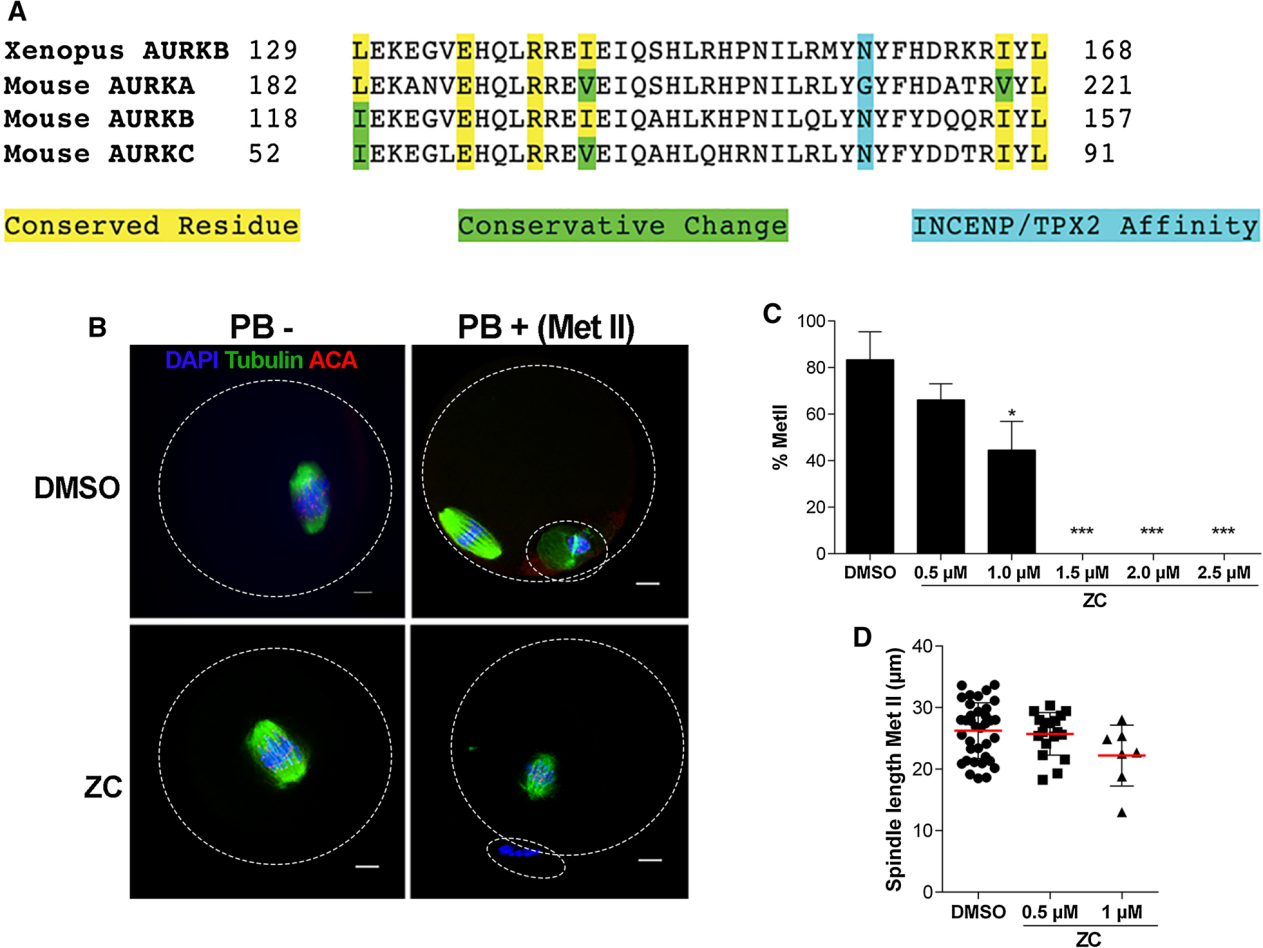
The ZINC08918027 (ZC) binding region is conserved, and the inhibitor perturbs meiotic progression in mouse oocytes. **A** Protein alignment of *Xenopus* AURKB and mouse AURKA/B/C. Yellow and green highlighted text indicate conserved residues and conservative changes in the ZC binding region respectively. The blue highlighted residues determine whether the AURK has affinity for INCENP (asparagine, N) or TPX2 (glycine, G). **B** Prophase I-arrested mouse oocytes were treated with the indicated doses of ZC and matured in vitro for 16 h, followed by detection of spindle (α-tubulin, green), kinetochores (anti-centromere antibody (ACA), red), and DNA (DAPI, blue). Met II progression was marked by polar body extrusion. The panels show representative images of metaphase I (Met I) arrest (PB −) and progression to metaphase II (Met II) (PB +). White circle outlines the oocyte and extruded polar bodies. Scale bar = 10 μm. **C** Percent of oocytes in each treatment that matured to the Met II stage. Error bars show standard error of the mean. **D** Quantification of Met II spindle length in each treatment. Data from three independent experiments with an average number of 25 Prophase I oocytes used/treatment/replicate (***p ≤ 0.001, One-way ANOVA)

**Fig. 2:**
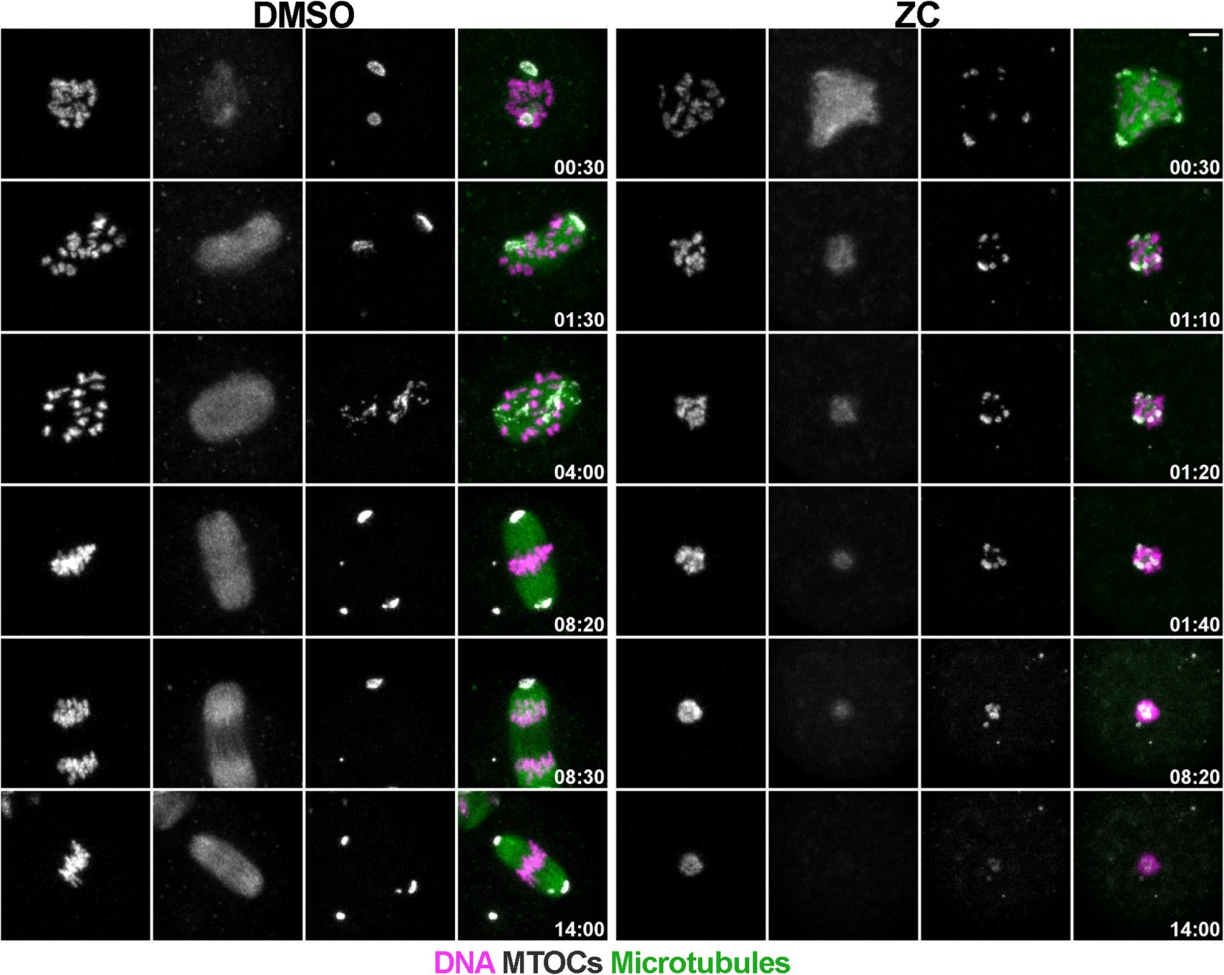
Dynamics of spindle formation in ZINC08918027 (ZC)-treated oocytes. Still images from movies were taken at 10-min intervals. Time 0:30 represents the start of imaging from Prophase I arrest release. Time when each image was taken is noted in white beneath each image. DNA (H2B-mCHERRY; magenta), microtubule organizing centers (MTOCs, CDK5RAP2-EGFP; white), and microtubules (fluorogenic dye SiR-tubulin; green). Data from 3 replicates with 10 oocytes/treatment/replicate

**Fig. 3:**
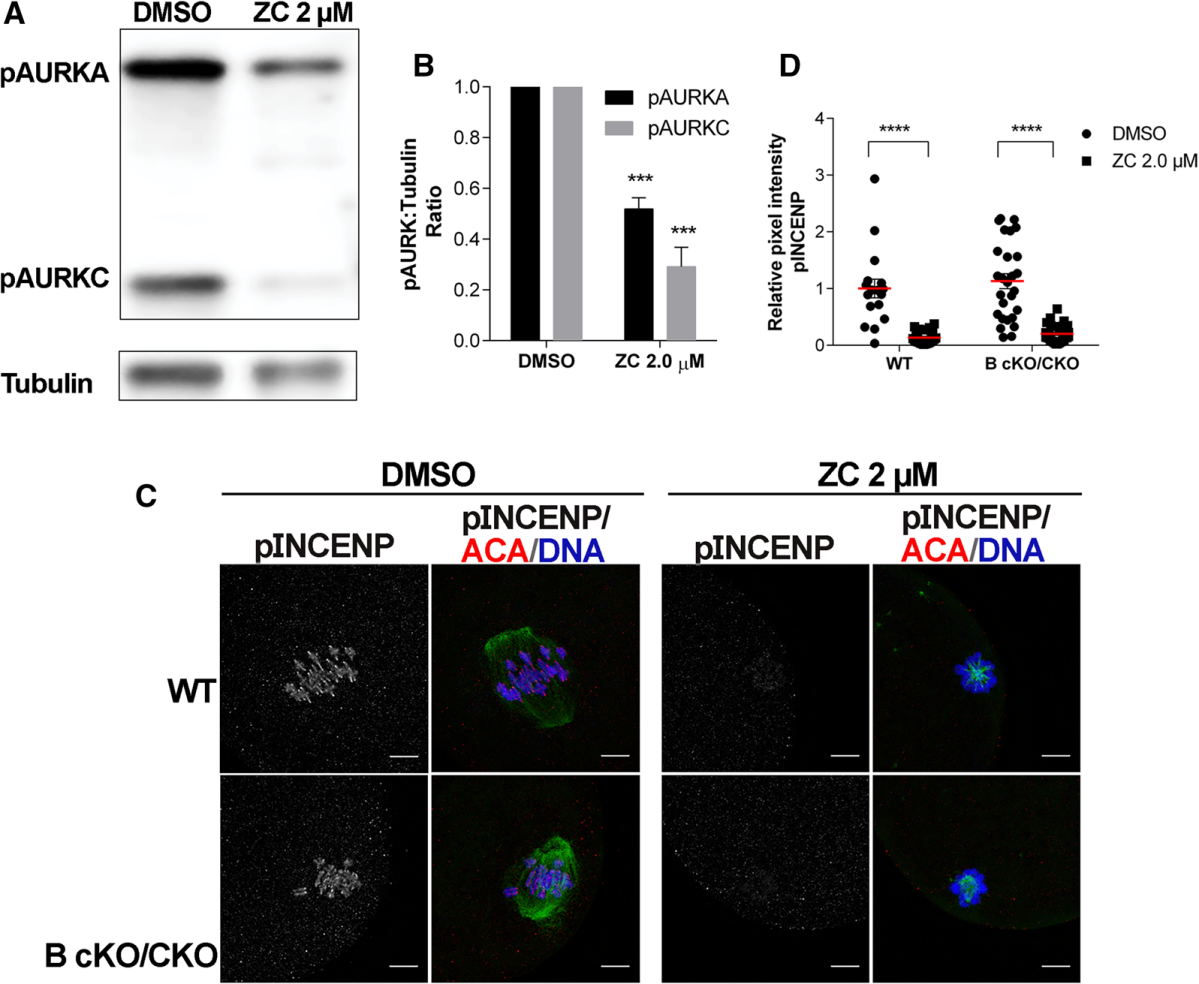
ZINC08918027 (ZC) can inhibit both AURKA and AURKC. Prophase I-arrested mouse oocytes were treated with the indicated doses of ZC and matured in vitro to Metaphase I before freezing (**A**, **B**) or fixation (**C**). **A** Oocyte lysates (100 oocytes/per lane) were separated via SDS-PAGE and transferred to a membrane for western blotting to detect the activated forms of AURKA (pAURKA) and AURKC (pAURKC). Tubulin was used as a loading control. **B** Quantification of pAURKA and pAURKC normalized to Tubulin. Error bars show standard error of the mean. Data from three independent experiments (***p ≤ 0.001, (Unpaired Students t-Test, two-tailed)). **C** Wild-type (WT) or oocytes lacking both AURKB and AURKC were probed with anti-phosphorylated INCENP (pINCENP) antibodies (gray), α-tubulin (green) and mounted in DAPI (blue). Scale bar = 10 μm. **D** Quantification of integrated density of pINCENP from (**C**). WT DMSO n = 17, WT Zn 2 μM = 25, B cKO/C KO DMSO n = 27, B cKO/C KO Zn 2 μM = 33

Female CF-1 mice (Envigo; Figs. 1, 3A, B) or CD-1 mice (Charles River Laboratories; Fig. 2 and Additional file 2: Video S1) between 6 and 8 weeks old were used for most experiments. Animals were euthanized via cervical dislocation without anesthesia to prevent stress hormone release. The generation and genotyping of *Aurkb* conditional knockout (cKO)/*Aurkc* knockout (KO) mice (Fig. 3C) were described previously [15]. Animals were housed in 12–12 h light–dark cycle, with constant temperature and with food and water provided ad libitum.

#### Oocyte collection and maturation

Female mice were injected intraperitoneally with 5 I.U. of pregnant mare's serum gonadotropin (Lee Biosolutions, # 493-10). Prophase I-arrested oocytes were collected from both ovaries 48 h post-injection; oocyte numbers depended upon the experiment (indicated in legends) whereas live imaging experiments used at least 20 oocytes/treatment/replicate and western blotting used 100 oocytes/treatment per replicate. Oocytes from multiple mice were mixed together prior to splitting into experimental and control groups to reduce animal-to-animal variation. 2.5 μM Milrinone (Sigma-Aldrich #M4659) was added to bicarbonate free minimal essential medium (MEM) (25 mM Hepes, pH 7.3, and 3 mg/mL polyvinylpyrrolidone) to prevent the oocytes from resuming meiosis spontaneously during collection. Oocytes were cultured in Chatot, Ziomek, and Bavister medium without milrinone at 37 °C in 5% CO_2_ [18]. For Metaphase I experiments, oocytes were cultured for 7.5 h. For Metaphase II experiments, oocytes were cultured for 16 h. Organ culture dishes (Becton Dickinson #353037) were used for in vitro maturation of drug-treated oocytes. At the end of the maturation period, the samples were fixed for immunostaining or frozen on dry ice for SDS-PAGE. All drug concentrations were prepared at a dilution factor of 1:2000.

For live-cell confocal imaging, prophase I-arrested oocytes were microinjected in M2 medium (Sigma-Aldrich) and cultured in MEM medium (Sigma-Aldrich) supplemented with 1.14 mM sodium pyruvate (Sigma-Aldrich), 4 mg/ml bovine serum albumin (Sigma-Aldrich), 75 U/ml penicillin (Sigma-Aldrich) and 60 μg/ml streptomycin (Sigma-Aldrich), at 37 °C in a 5% CO_2_. Oocytes were stained with 100 nM SiR-tubulin (Spirochrome) for microtubule visualization. After removing the cumulus cells, oocytes were microinjected in M2 medium with ~ 10 pl of 50 ng/μl H2b-mCherry, and 125 ng/μl Egfp-Cdk5rap2. Microinjected oocytes were cultured for 3 h in MEM medium supplemented with milrinone to allow protein expression prior to experimental procedures.

See video protocol [19] for detailed protocol methods for collection, injections and immunocytochemistry.

#### Plasmids

To generate cRNAs, plasmids were linearized and in vitro transcribed using a mMessage mMachine T3 (Ambion #AM1348) and T7 kits (Ambion #AM1344), according to manufacturer’s protocol. The synthesized cRNAs were then purified using an RNAeasy kit (Qiagen #74104) and stored at − 80 °C. *H2B-mCherry* and *mEGFP-Cdk5rap2* cRNA constructs were described previously [20].

#### Western blotting

Oocytes were cleaned of cumulus cells with repetitive pipetting. Metaphase I oocytes (100/treatment) were mixed with sample buffer (1% SDS, 1% β-mercaptoethanol, 20% glycerol, 50 mM Tris–HCl (pH 6.8)) and phosphatase inhibitors sodium fluoride (25 mM) and sodium orthovanadate (1 mM) and denatured at 95 °C for 5 min. Western blotting for pAURKA/B/C (1:500; Cell Signaling Technologies #2914) was conducted as detailed previously [7, 21]. The rabbit Tubulin antibody (Cell Signaling Technology, #11H10) was used as a loading control.

#### Immunocytochemistry

Oocytes were fixed in phosphate-buffered saline containing 2% paraformaldehyde (PFA; Sigma-Aldrich #P6148) for 20 min. Permeabilization and staining was conducted as described previously [19]. After final washes, the cells were mounted in VectaShield (Vector Laboratories, #H-1000, Burlingame, USA) with 4′, 6-Diamidino-2-Phenylindole, Dihydrochloride (DAPI; Life Technologies #D1306; 1:170).

#### Antibodies

For immunofluorescence experiments, the primary antibodies were as follows: mouse anti α-tubulin Alexa-fluor 488 conjugated (1:100, Life Technologies #322588), rabbit anti-pINCENP (1:1000, gift from M. Lampson, University of Pennsylvania; [22]) and anti-centromeric antigen (ACA) (1:30, Antibodies Incorporated #15-234). The following secondary antibodies were used for immunofluorescence at a concentration of 1:200: anti-rabbit-Alexa568 (Life Technologies #A11011) and goat anti-human-Alexa 568 (Life Technologies #A21090).

#### Microscopy

A Leica TCS SP8 confocal microscope, using a 40 × objective, was used to capture the images. Optical z-slices were obtained separately for each image using a 1.0 μm step and a zoom setting of 3.5. The power of the laser was left unchanged for each cell if pixel intensities were to be compared. Samples were coded so that images and analysis of treatment groups were obtained and conducted in a blinded fashion.

#### Live cell imaging

Time-lapse image acquisition was performed using a Leica TCS SP5 microscope with an HCX PL Apo Lambda Blue 40 × 1.25 NA oil objective. Oocytes were scanned using sequential scan in between line mode at a 12-bit image depth with 7.75 × zoom on the chromosome area. 3D scanning was performed using 2.5-µm optical sections through spindle volume. Image analysis was performed using FiJi software [23].

#### Statistical analysis

To evaluate differences between and among groups, a one-way analysis of variance (ANOVA) was used on Prism software (GraphPad). For all experiments, significance was marked by a p-value less than or equal to 0.05. Experiments were performed in triplicate and oocyte number per replicate and/or total number is indicated in the figure legends.

#### ZINC08918027 binding site sequence alignment

The sequences of *Xenopus laevis* AURKB (accession number: AAM76715), *Mus musculus* AURKB (accession number: AAH03261), *Mus musculus* AURKC (accession number: AAH64780), and *Mus musculus* AURKA (accession number: AAH14711) were aligned using the MacVector with Assembler software (MacVector, Inc.).

## Results and discussion

Because all three AURK isoforms can bind INCENP [16, 24, 25], we hypothesized that ZC could block the interaction between any AURK and INCENP in a cell type that expresses different AURK-containing CPC forms. ZC was designed to target the INCENP binding site on *Xenopus laevis* AURKB [17]. Therefore, to explore the similarities of this region between organisms, we aligned the ZC-binding region of *Xenopus* AURKB with the corresponding region in each mouse AURK. The alignment revealed that the ZC-binding region was conserved in all three mouse isoforms (Fig. 1A). Residues in this binding region were either identical or were a biochemically conservative change. These analyses support our hypothesis that ZC can prevent binding between INCENP and any AURK isoform.

We then sought to determine the optimal concentration of ZC to use in mouse oocytes. The CPC is important for cytokinesis [26]. Therefore, we aimed to determine the lowest concentration of ZC at which polar body extrusion (PBE), the asymmetric cytokinesis in oocytes, was abolished indicating CPC disruption. We performed a dose–response curve and matured oocytes in vitro to Metaphase II (Met II) (Fig. 1B). The results showed that PBE decreased in a dose-dependent manner, with complete PBE failure beginning in 1.5 μM ZC (Fig. 1C). In 1 μM ZC, PBE was reduced by > 50% (Fig. 1C). The spindles of ZC-treated Met II eggs that did extrude polar bodies appeared shorter in length than the spindles of dimethylsulfoxide (DMSO)-treated oocytes (Fig. 1B, D). This finding is consistent with inhibition of AURKC-CPC and when AURKA is deleted [7, 9].

Because the spindle lengths of ZC-treated oocytes were shorter (Fig. 1D), we hypothesized that ZC adversely impacts spindle formation. To test this hypothesis, we conducted live imaging of oocyte maturation in the presence of ZC and chose a dose (2.5 μM) where all oocytes failed to extrude a polar body (Fig. 1C). In control oocytes, the microtubule organizing centers (MTOCs) fragmented after nuclear envelope breakdown (NEBD), then clustered into two spindle poles before the chromosomes aligned at the metaphase plate, consistent with prior studies [27]. On the contrary, we observed that 100% of the oocytes treated with ZC never formed a bipolar spindle. We observed a disappearance of microtubule signal, and MTOCs gradually approached each other and moved towards the center of the oocyte. Furthermore, chromosomes did not align at the metaphase plate and instead condensed into a “ring-like” structure surrounding the cluster of MTOCs (Fig. 2; Additional file 2: Video S1). Therefore, we concluded that ZC disturbed spindle building in mouse oocytes. Taken together, these data demonstrate that ZC disrupts the formation of a bipolar spindle in mouse oocytes, which contributes to the reduction in PBE rates observed with ZC treatment.

ZC was designed with the intention to create an AURKB-specific inhibitor by blocking INCENP binding. However, because of the high degree of sequence conservation in this AURK domain (Fig. 1A) and because AURKA can bind INCENP in oocytes, we hypothesized that in oocytes ZC is not AURKC-specific and inhibits AURKA. To test this hypothesis, we first assessed AURKA/C activation. We treated WT oocytes with 2 μM ZC and separated proteins from cell lysates by SDS-PAGE to resolve the activated forms of AURKA and AURKC from one another. In control-treated oocytes, activated AURKA (pAURKA) is more abundant than activated AURKC (pAURKC) (Fig. 3A, Additional file 1: Fig. S1). Upon ZC treatment, pAURKC declines ~ 80% (Fig. 3A, B), suggesting that both INCENP and non INCENP-bound populations of AURKC exist in oocytes. Importantly, we also saw a decline in pAURKA by ~ 50% supporting the hypothesis that some AURKA is INCENP-bound in WT mouse oocytes. To further support this hypothesis, we used oocytes from mice lacking both *Aurkb* and *Aurkc*. We previously showed that in these double knockout oocytes, AURKA localizes to chromosomes, phosphorylates INCENP, and compensates for *Aurkb*/*c* loss [15]. These oocytes provide a simplified genetic background in which to visualize the impact of ZC on AURKA-CPC activity. To visualize AURKA-CPC activity, we probed oocytes with a phospho-specific INCENP antibody that detects the Aurora kinase specific phosphorylation sites [22]. WT and double knockout oocytes treated with DMSO contained similar levels phosphorylated INCENP when we examined chromosomes at Met I (Fig. 3C, D). When WT oocytes were treated with 2 μM ZC, pINCENP immunoreactivity was not detected. Similarly, pINCENP was absent when double *Aurkb*/*c* knockout oocytes with were treated with 2 μM ZC (Fig. 3C, D). Taken together, these data indicate that ZC can prevent both AURKA and AURKC from binding INCENP and suggest that WT oocytes possess AURKA-CPC that is not visibly detectable, like some mitotic cell lines.

### Limitations

Because oocytes are limiting in number, our studies are limited to assessing change in activity by immunofluorescence and western blotting. Therefore, we cannot isolate AURKA-INCENP and AURKA-TPX2 from oocytes to conduct biochemical analyses. Mouse oocytes also express AURKB, which is much less abundant and more challenging to detect. In our assays, we have not determined the impact ZC has on AURKB and this impact remains an open question. We also do not know if these results translate to human oocytes.

## Supplementary Information

**Additional file 1: Figure S1.** Full western blot image detecting the activated forms of AURKA (pAURKA) and AURKC (pAURKC) (top) and alpha-tubulin (bottom) from oocytes treated with ZC and matured to Met I. Red box: Area of cropping shown in Fig. 3A. Experiment repeated 3 times.

**Additional file 2: Video S1.** Dynamics of spindle formation in control and ZINC08918027 (ZC) treated oocytes. Time 0:00 represents the start of maturation from Prophase I arrest. DNA (H2B-mCHERRY), microtubule organizing centers (MTOCs, CDK5RAP2-EGFP), and microtubules (fluorogenic dye SiR-tubulin). N = 10 oocytes/treatment.

## Data Availability

The imaging datasets used and/or analysed are available from the corresponding author upon request.

## Abbreviations

ACA: Anti-centromeric antigen
AURK: Aurora kinase
cKO: Conditional knockout
CPC: Chromosomal passenger complex
DMSO: Dimethylsulfoxide
KO: Knockout
MEM: Minimal essential medium
MTOC: Microtubule organizing center
NEBD: Nuclear envelope breakdown
PBE: Polar body extrusion
ZC: ZINC08918027

## References

[1] Carmena M, Earnshaw WC (2003). The cellular geography of aurora kinases. Nat Rev Mol Cell Biol.

[2] Willems E, Dedobbeleer M, Digregorio M, Lombard A, Lumapat PN, Rogister B (2018). The functional diversity of Aurora kinases: a comprehensive review. Cell Div.

[3] Brown JR, Koretke KK, Birkeland ML, Sanseau P, Patrick DR (2004). Evolutionary relationships of Aurora kinases: implications for model organism studies and the development of anti-cancer drugs. BMC Evol Biol.

[4] Seeling JM, Farmer AA, Mansfield A, Cho H, Choudhary M (2017). Differential selective pressures experienced by the Aurora kinase gene family. Int J Mol Sci.

[5] Yao LJ, Zhong ZS, Zhang LS, Chen DY, Schatten H, Sun QY (2004). Aurora-A is a critical regulator of microtubule assembly and nuclear activity in mouse oocytes, fertilized eggs, and early embryos. Biol Reprod.

[6] Saskova A, Solc P, Baran V, Kubelka M, Schultz RM, Motlik J (2008). Aurora kinase A controls meiosis I progression in mouse oocytes. Cell Cycle.

[7] Blengini CS, Ibrahimian P, Vaskovicova M, Drutovic D, Solc P, Schindler K (2021). Aurora kinase A is essential for meiosis in mouse oocytes. PLoS Genet.

[8] Yang KT, Li SK, Chang CC, Tang CJ, Lin YN, Lee SC (2010). Aurora-C kinase deficiency causes cytokinesis failure in meiosis I and production of large polyploid oocytes in mouse. Mol Biol Cell.

[9] Balboula AZ, Schindler K (2014). Selective disruption of aurora C kinase reveals distinct functions from aurora B kinase during meiosis in mouse oocytes. PLoS Genet.

[10] Kelly AE, Funabiki H (2009). Correcting aberrant kinetochore microtubule attachments: an Aurora B-centric view. Curr Opin Cell Biol.

[11] Santaguida S, Vernieri C, Villa F, Ciliberto A, Musacchio A (2011). Evidence that Aurora B is implicated in spindle checkpoint signalling independently of error correction. EMBO J.

[12] Nezi L, Musacchio A (2009). Sister chromatid tension and the spindle assembly checkpoint. Curr Opin Cell Biol.

[13] Blengini CS, Nguyen AL, Aboelenain M, Schindler K (2021). Age-dependent integrity of the meiotic spindle assembly checkpoint in females requires Aurora kinase B. Aging Cell.

[14] Aboelenain M, Schindler K (2021). Aurora kinase B inhibits aurora kinase A to control maternal mRNA translation in mouse oocytes. Development.

[15] Nguyen AL, Drutovic D, Vazquez BN, El Yakoubi W, Gentilello AS, Malumbres M (2018). Genetic interactions between the aurora kinases reveal new requirements for AURKB and AURKC during oocyte meiosis. Curr Biol.

[16] DeLuca KF, Meppelink A, Broad AJ, Mick JE, Peersen OB, Pektas S (2018). Aurora A kinase phosphorylates Hec1 to regulate metaphase kinetochore-microtubule dynamics. J Cell Biol.

[17] Unsal E, Degirmenci B, Harmanda B, Erman B, Ozlu N (2016). A small molecule identified through an in silico screen inhibits Aurora B-INCENP interaction. Chem Biol Drug Des.

[18] Chatot CL, Ziomek CA, Bavister BD, Lewis JL, Torres I (1989). An improved culture medium supports development of random-bred 1-cell mouse embryos in vitro. J Reprod Fertil.

[19] Stein P, Schindler K (2011). Mouse oocyte microinjection, maturation and ploidy assessment. J Vis Exp.

[20] Balboula AZ, Nguyen AL, Gentilello AS, Quartuccio SM, Drutovic D, Solc P (2016). Haspin kinase regulates microtubule-organizing center clustering and stability through Aurora kinase C in mouse oocytes. J Cell Sci.

[21] Fellmeth JE, Gordon D, Robins CE, Scott RT, Treff NR, Schindler K (2015). Expression and characterization of three Aurora kinase C splice variants found in human oocytes. Mol Hum Reprod.

[22] Salimian KJ, Ballister ER, Smoak EM, Wood S, Panchenko T, Lampson MA (2011). Feedback control in sensing chromosome biorientation by the Aurora B kinase. Curr Biol.

[23] Schindelin J, Arganda-Carreras I, Frise E, Kaynig V, Longair M, Pietzsch T (2012). Fiji: an open-source platform for biological-image analysis. Nat Methods.

[24] Abdul Azeez KR, Chatterjee S, Yu C, Golub TR, Sobott F, Elkins JM (2019). Structural mechanism of synergistic activation of Aurora kinase B/C by phosphorylated INCENP. Nat Commun.

[25] Katayama H, Sasai K, Kloc M, Brinkley BR, Sen S (2008). Aurora kinase-A regulates kinetochore/chromatin associated microtubule assembly in human cells. Cell Cycle.

[26] Hadders MA, Lens SMA (2022). Changing places: chromosomal passenger complex relocation in early anaphase. Trends Cell Biol.

[27] Clift D, Schuh M (2015). A three-step MTOC fragmentation mechanism facilitates bipolar spindle assembly in mouse oocytes. Nat Commun.

