# Supplementary material for: Using ZINC08918027 inhibitor to determine Aurora kinase-chromosomal passenger complex isoforms in mouse oocytes
Source: BMC Res Notes. 2022 Mar 7;15:96. doi: 10.1186/s13104-022-05987-4 (PMC8900367; doi:10.1186/s13104-022-05987-4)
Supplement: Supplementary file 1 — Additional file 1: Figure S1. Full western blot image detecting the activated forms of AURKA (pAURKA) and AURKC (pAURKC) (top) and alpha-tubulin (bottom) from oocytes treated with ZC and matured to Met I. Red box: Area of cropping shown in Fig. 3A. Experiment repeated 3 times. [file 13104_2022_5987_MOESM1_ESM.docx]

Kratka et al.

**Supplemental Figure legends**

**Figure S1.** Uncropped western blot detecting the activated forms of AURKA (pAURKA) and AURKC (pAURKC) (top) and alpha-tubulin (bottom) from oocytes treated with ZC and matured to Met I. Red box: Area shown in Fig 3A. Experiment repeated 3 times.


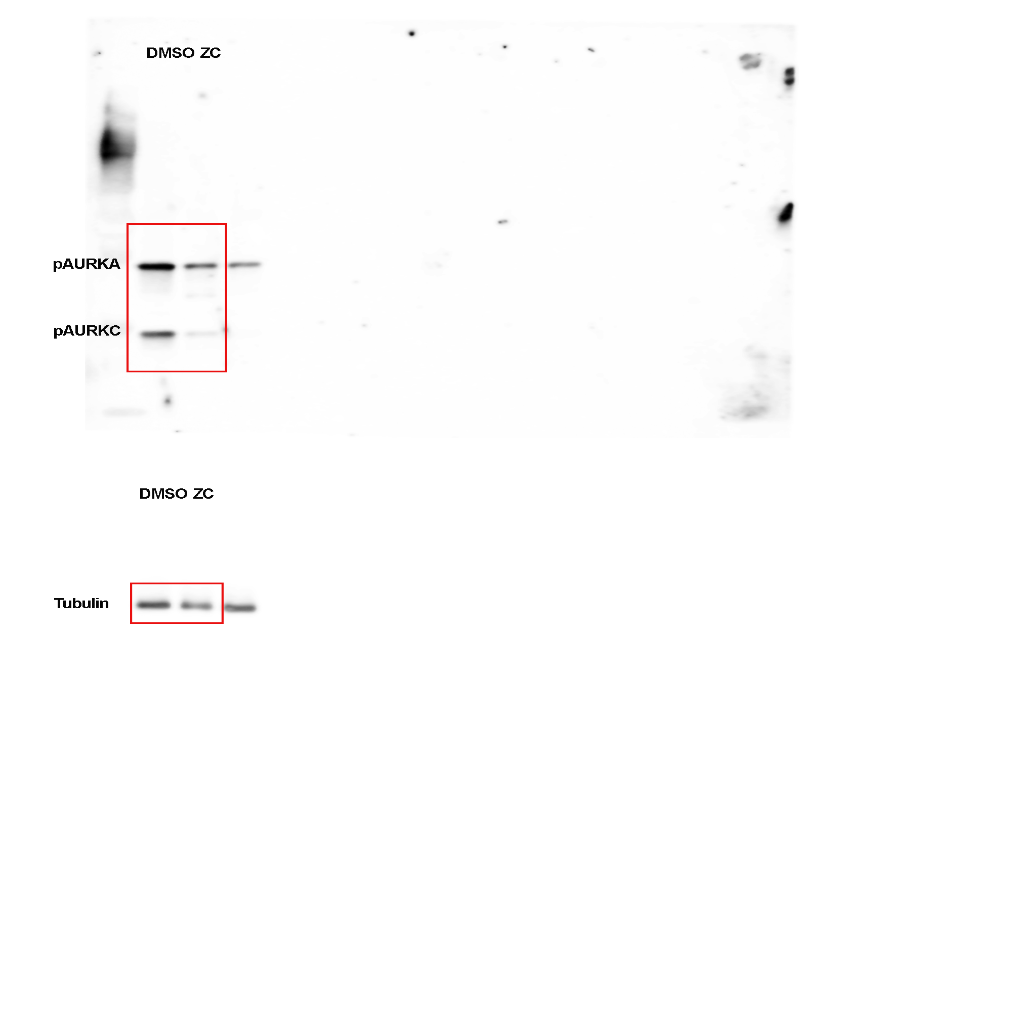


**Video S1.** Dynamics of spindle formation in control and ZINC08918027 (ZC) treated oocytes. Time 0:00 represents the start of maturation from Prophase I arrest. DNA (H2B-mCHERRY), microtubule organizing centers (MTOCs, CDK5RAP2-EGFP), and microtubules (fluorogenic dye SiR-tubulin). N = 10 oocytes/treatment
